# p53 negatively regulates the osteogenic differentiation of vascular smooth muscle cells in mice with chronic kidney disease

**DOI:** 10.5830/CVJA-2011-069

**Published:** 2012-07

**Authors:** KL Li, ZH Li, J Zhan, YN He, J Chen, L Zhao

**Affiliations:** Department of Nephrology, Research Institute of Surgery, Daping Hospital, Third Military Medical University, Chongqing, China; Department of Nephrology, Research Institute of Surgery, Daping Hospital, Third Military Medical University, Chongqing, China; Department of Nephrology, Research Institute of Surgery, Daping Hospital, Third Military Medical University, Chongqing, China; Department of Nephrology, Research Institute of Surgery, Daping Hospital, Third Military Medical University, Chongqing, China; State Key Laboratory of Trauma, Burn and Combined Injury, Research Institute of Surgery, Daping Hospital, Third Military Medical University, Chongqing, China; State Key Laboratory of Trauma, Burn and Combined Injury, Research Institute of Surgery, Daping Hospital, Third Military Medical University, Chongqing, China

**Keywords:** chronic kidney disease, mouse, osteogenic differentiation, P53, vascular smooth muscle cells

## Abstract

**Aim:**

To investigate the osteogenic differentiation of vascular smooth muscle cells (VSMCs) in mice with chronic kidney disease (CKD) and to evaluate the effects of p53 on the osteogenic differentiation of the VSMCs.

**Methods:**

Experimental models of CKD-associated vascular calcification generated by five-sixth (5/6) nephrectomy (Nx) and a high-phosphate (HP) diet were used in p53+/+ and p53–/– mice. Following 5/6 Nx, aortic calcification, markers of osteogenic differentiation, VSMCs and p53 protein in aortic tissues were studied.

**Results:**

Aortic calcification was observed after eight weeks following 5/6 Nx in mice of both genotypes, and expression of the markers of osteogenic differentiation in the VSMCs was increased. These changes were continuously observed up to 12 weeks after 5/6 Nx, and particularly after 5/6 Nx + HP. Compared with p53+/+ mice, aortic calcification in p53–/– mice was more severe (*p* < 0.001). Expression of the markers of osteogenic differentiation was noticeably increased (*p* < 0.001), while expression of the marker of VSMCs had decreased (*p* < 0.001). Statistical analysis demonstrated that the markers of osteogenic differentiation were negatively correlated with p53, and the marker of VSMCs was positively correlated with p53 (*p* < 0.001).

**Conclusion:**

p53 has the potential to negatively regulate the osteogenic differentiation of VSMCs in CKD mice.

## Abstract

It is increasingly apparent that individuals with chronic kidney disease (CKD) are more likely to die of cardiovascular disease (CVD) than to develop kidney failure,^1^,^2^ and CVD accounts for approximately 50% of the premature deaths in dialysis patients.^3^,^4^ Vascular calcification is more prevalent and more severe in patients with stage V CKD (CKD-V),^5^ and the extent of vascular calcification has been identified as an independent risk factor for cardiovascular death in patients on haemodialysis.^3^ Calcification can be found in atherosclerotic plaques and in the vascular media, vascular smooth muscle cells (VSMCs) and elastic laminae of large elastic and medium muscular arteries, as well as in the cardiac valves.^1^,^6^

Recent evidence indicates that vascular calcification is an active, cell-mediated process. Osteoblast differentiation core-binding factor α-1 (RUNX2) and several bone-associated proteins, such as bone morphogenetic protein-2 (BMP-2) and osterix (Osx), and alkaline phosphatase (ALP) are present in histological sections of arteries as well as in uraemic serum obtained from patients with CKD-V. This supports the theory that VSMCs can dedifferentiate or transform into osteoblast-like cells. The dedifferentiation of VSMCs via up-regulation of core-binding factor a1 (Cbfa1) may be the first step in the process of calcification of the arteries.^5^,^7^,^8^ In addition, it is likely that circulating inhibitors of calcification are also important, but to date, the process is not clearly understood, particularly the effect of cell cycle regulatory proteins (p53 for example) on the osteogenic differentiation of VSMCs.

Activation of the tumour suppressor p53 induces cellular programmes, including cell cycles, to shut down and undergo apoptosis or senescence, which prevents the accumulation of genetically altered cells.^9^-^11^ Recent studies have found that p53 plays a critical role in bone organogenesis and homeostasis by negatively regulating bone development and growth and by suppressing bone neoplasia. Murine double-minute (Mdm2)-mediated inhibition of p53 function is a prerequisite for RUNX2 activation, osteoblast differentiation and proper skeletal formation.^12^ In atherosclerosis, p53 not only arrests growth and promotes cell senescence and apoptosis, but also protect against trans-differentiation of bone marrow stromal cells into VSMCs, protects against apoptosis, and alters the mode of cell death within the plaque.

In a previous study, we found that vascular calcification was extensively present in patients with CKD-V on maintenance haemodialysis, and it was accompanied by decreased expression of p53 in VSMCs. This result indicates that inhibition of expression p53 in VSMCs may be involved in the pathogenesis of vascular calcification.^13^ To further investigate the effects of p53 on the osteogenic differentiation of VSMCs, we used wild-type (p53+/+) and p53-deficient (p53–/–) mice in this study to construct animal models of CKD-associated vascular calcification. Some of the mice were subjected to five-sixth (5/6) nephrectomy (Nx) and a high-phosphate (HP) diet. At different time points following 5/6 Nx, we studied histological changes in the kidney, aortic calcification, the markers of osteogenic differentiation (such as BMP-2, RUNX2, Osx and ALP), a special marker of VSMCs [alpha smooth muscle actin (α-SMA)], and the expression of p53 protein in the aortic tissue. The effects of p53 on the osteogenic differentiation of VSMCs were evaluated.

## Methods

p53–/– mice (F2) in C57BL/6 background mice (F2) were obtained from the NIH and housed in a pathogen-free environment. p53+/– mice were crossed with p53+/– mice to generate homozygous p53–/– and p53+/+ littermates. Genotyping of the mice was performed by polymerase chain reaction analysis of DNA extracted from the tail tips, as reported by Donehower.^10^,^14^ Male p53–/– and p53+/+ mice aged two months were used in this study.

The experiments were carried out in accordance with the Research Council and Animal Care and Use Committee of the Research Institute of Surgery, Daping Hospital, Third Military Medical University (Chongqing, China). Experiments conformed to the guidelines of the ethical use of animals. The animal study protocol was reviewed and approved by the Animal Ethics Committee of Chongqing. Efforts were made to minimise animal suffering and the number of mice used.

We used a well-established murine model of a CKD-associated vascular calcification model. Briefly, male mice weighing 19 to 24 g were housed with a 12-hour light and dark cycle and allowed free access to food and water. All animals were anesthetised with sodium pentobarbital (50 mg/kg i.p.) and then placed on a warming table to maintain a rectal temperature of 37°C.

5/6 Nx as a model of CKD was achieved in a two-step surgical procedure.^3^,^15^,^16^ The left kidney was exposed by a flank incision and the upper and lower poles of the right kidney were resected. Two weeks later the left kidney was removed. The mouse was allowed to recover in a warmed cage, and food and water were given *ad libitum*. The day after the 5/6 Nx, the animals received standard HP diet (Altromin C1049, Germany, containing 1.65% phosphate, 0.24% sodium, 0.95% calcium, 0.07% magnesium, 0.7% potassium and 17% protein).

## Experimental design

Mice were randomly assigned to six experimental groups: (1) p53+/+ mice sham-operated on a normal diet (0.6% Ca and 0.6% P) were used as the control (group 1, *n* = 10) with the p53+/+ group, (2) p53–/– mice sham-operated on a normal diet were used as the control (group 2, *n* = 10) with the p53–/– group, (3) 5/6 Nx of p53+/+ mice on a normal diet (group 3, *n* = 10), (4) 5/6 Nx of p53–/– mice on a normal diet (group 4, *n* = 10), (5) 5/6 Nx of p53+/+ mice + HP diet (group 5, *n* = 10), and (6) 5/6 Nx of p53–/– mice + HP diet (group 6, *n* = 10).

Food consumption in groups 1 to 4 was the same, and that in groups 5 and 6 was the same. The high mortality in 5/6 Nx mice precluded maintaining them for more than 12 weeks in this study. At eight and 12 weeks following 5/6 Nx, the mice were anaesthetised with intraperitoneal pentobarbital (five animals at each time point in each group). Blood was collected by retro-orbital bleeding. The mice were killed by cervical dislocation and the kidneys and aortic tissue were collected. Each kidney or aorta was cut into three parts medially for the following analyses.

## Analyses

Paraffin-embedded sections of 4 μm were prepared and stained with haematoxylin and eosin (HE) stain and then examined in a blinded manner by two examiners, with each section evaluated twice. Glomerular cell number was determined by counting the nuclei within the glomerular tuft. Glomerular sclerosis was graded as follows: 0 = none; +1 = sclerotic changes in < 25% of the glomerulus; +2 = 25– 50% sclerosis; +3 = > 50% sclerosis.^17^ The mean score per glomerulus in each kidney was determined as the sclerosis index.

Tubulo-interstitial fibrosis was defined as tubular atrophy, dilation and intratubular casts, as well as cellular infiltration and widening of the interstitium. It was scored semi-quantitatively according to the method of Shih *et al*.^18^ as follows: 0 = normal; 0.5 = small focal area injured; 1 = less than 10% of the cortex injured; 2 = 10–25% of the cortex injured; 3 = 25–75% of the cortex injured; and 4 = > 75% of the cortex injured.

Measurements of body weight and blood pressure, and the levels of haemoglobin, blood urea nitrogen (BUN), phosphate (Pi) and calcium (Ca) were performed as described by Bro *et al*.^17^ Parathyroid hormone (PTH) level was determined by a commercial ELISA test (BioSource, Belgium).

Mineral deposition (calcification) was assessed under the light microscopic using the von Kossa assay. Dewaxed and rehydrated sections (4 μm) of artery tissue were placed in 5% silver nitrate solution for 30 min in the dark, then into revelator solution (Kodak) for 5 min. They were fixed in 5% sodium-thiosulfate solution for another 5 min. Finally the sections were counterstained with 2% eosin.

Calcium deposits appeared as black areas.^19^,^20^ For the vascular calcification score, sections were graded from 0 to 4+ for von Kossa staining,^21^ where 0 = no calcification; 1 = spots; 2 = single segments of black staining; 3 = multiple segments; and 4 = diffuse, circumferential staining. The calcification score was obtained by averaging all the scores from all sections.

The determination of BMP-2, RUNX2 and Osx in the aortic tissue was performed on cryostat sections (4 μm) using indirect immunofluorescence staining. Briefly, the sections were fixed with 4% formaldehyde/PBS (pH 7.4) and treated with 3% H_2_O_2_ in methanol for 10 min to inactivate endogenous peroxidase. After washing in PBS, the sections were microwaved in 10 mM citrate buffer (pH 6.0) for 10 min to retrieve the antigen. Sections were then incubated with 1.5% normal goat serum for 15 min, followed by incubation with primary antibodies, BMP-2 antibody (1:200, code: sc-6895, Santa Cruz, USA), RUNX2 antibody (1:200, code: sc-12488, Santa Cruz, USA), or Osx antibody (1:200, code: sc-22536-R, Santa Cruz, USA) at 37°C for three hours.

After removal of the unbound primary antibody and rinsing with PBS, the sections were incubated with fluorescein isothiocyanate-conjugated (FITC) antibody (code: AP186F, Chemicon, Germany) diluted 1:100 in PBS at 37°C for 30 min. The slides were washed three times with PBS and stained with propidium iodide (Sigma) and Hoechst 33342 (Sigma). Negative controls consisted of substituting the primary antibody with PBS. Immunofluorescent images were visualised under a confocal microscope (LSM 510 META, Carl Zeiss, Jena, Germany). Positive results were identified by a green fluorescence.

Immunostaining for p53, ALP and α-SMA in the aorta tissue was performed on cryostat sections (4 μm) using the standard avidin–biotin complex method. Sections were processed as previously described.^10^ Anti-mouse p53 polyclonal antibody (1:200, code: SC-6243, Santa Cruz, USA), anti-ALP (1:200, GmbH, Germany) and monoclonal mouse anti-α-SMA (1:200, Sigma-Aldrich Co, St. Louis, MO, USA) were used as primary antibodies. Sections were incubated with primary antibody at 37°C for three hours.

After removal of unbound primary antibody and rinsing with PBS, the sections were incubated with avidin-biotinylated horseradish peroxidase (code: pk-7200, Vectastain Elite ABC kit; Vector Laboratories) for 60 min. The slides were washed again in PBS, then visualisation was performed using the diaminobenzidine (DAB) substrate–chromogen system (code: 3468, Dako, Glostrup, Denmark) and counterstained with 1% methylene green. Finally, sections were washed with tap water, dehydrated and mounted. Negative controls consisted of substituting the primary antibody with PBS. A brown colour indicated a positive result.

The Western blot analysis was carried out for determination of p53, BMP-2, RUNX2, Osx, ALP and α-SMA in the aortic tissue. Pieces of dissected aortic tissue were immersed in lysis buffer (50 mM Tris-HCl, pH 7.4, 0.1% SDS, 1% NP-40, 0.5% sodium deoxycholate, 100 mM NaCl, 0.1 mM sodium orthovanadate, 1 mM sodium fluoride, 10 μg/ml aprotinin, 10 μg/ml leupeptin, 10 μg/ml pepstatin, and 10 μg/ml PMSF), homogenised with a dounce homogeniser, and then incubated on ice for 30 min. After centrifuging at 20 000 × g and 4°C for 30 min, the protein concentration of the collected supernatant was determined by BCA kit (code: 23250, Pierce, Rockford, IL).

Samples of 40-μg protein were mixed with two × loading buffer containing 125 mM Tris-HCl (pH 6.8), 5% glycerol, 2% sodium dodecyl sulfate (SDS), 2% β-mercaptoethanol and 0.001% bromophenol blue and were electrophoresed on 13% sodium dodecyl sulfate-polyacrylamide gels for BMP-2 and ALP, or on 10% gels for p53, RUNX2, Osx and α-SMA. The proteins were transferred overnight to polyvinylidine difluoride (PVDF) membranes (Millipore, Bedford, MA) using a Bio-Rad Western blot apparatus.

After the proteins were transferred, the blots and gels were stained with Coomassie blue to check for complete protein transference and equal loading. Membranes were blocked in 5% skimmed milk and hybridised to the following primary antibodies against p53, BMP-2, RUNX2, Osx, ALP and α-SMA as mentioned above (1:2 000) in anti-glyceraldehyde-3-phosphate dehydrogenase (GAPDH) mAb (1:2 000; code: sc-47724, Santa Cruz, USA). The membranes were washed in 20 mM Tris, pH 7.5, 150 mM NaCl, 0.05% (v/v) Tween-20, and hybridised to the corresponding horseradish peroxidase-conjugated secondary antibodies, goat anti-rabbit IgG-HRP (code: SC-2004, Serologicals Corporation) 1:3 000 or goat anti-mouse IgG-HRP (code: 12-349, Serologicals Corporation) 1:3 000 at room temperature for one hour.

Chemiluminescence detection was performed using Supersignal (code: 34080, Pierce, IL, USA) according to the manufacturer’s instructions and images were acquired on X-ray film. Quantitative densitometry was performed on the identified bands using a computer-based measurement system.

## Statistical analysis

Results are expressed as mean ± SEM. Data analysis was performed using SPSS (SPSS Inc, Chicago, IL, Version 11.0). Means of total cell number per glomerulus, glomerular sclerosis index score and tubulo-interstitial fibrosis index score (histological index score), as well as body weight, blood pressure and serum chemistry parameters in non-size matched experimental groups were compared using Kruskal-Wallis non-parametric tests.

To determine the differences in vascular calcification and the expression levels of the proteins in p53+/+ and p53–/– mice at different time points after 5/6 Nx, the Fisher exact test was used. For calculating the correlation between the expression of p53 protein and vascular calcification or osteogenic differentiation proteins, linear correlation analysis was done on the Western blot data. Statistical significance was defined as *p* < 0.05.

## Results

**Fig. 1.**
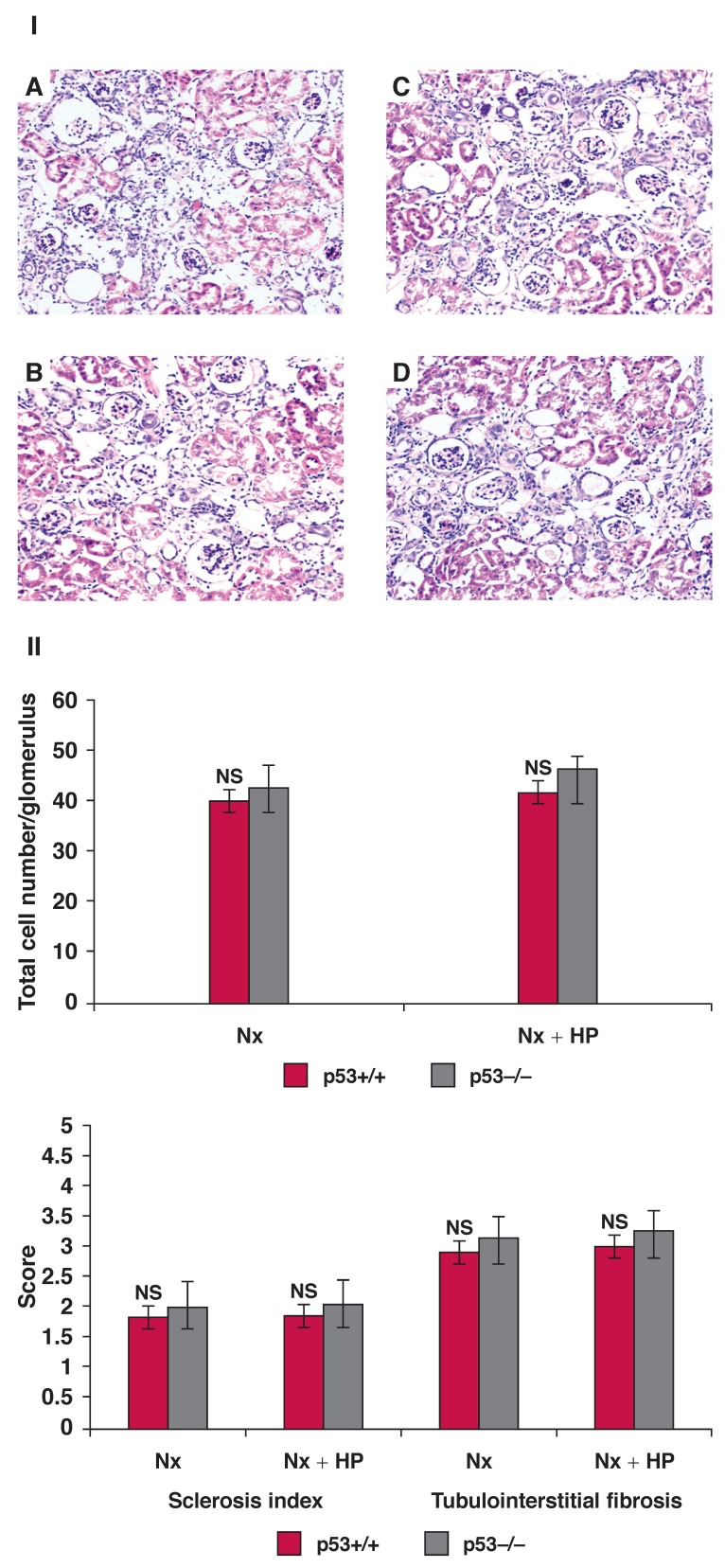
I: Histological appearance of the kidney in A and C: p53+/+ mice, and B and D: p53–/– mice at 12 weeks after 5/6 Nx or 5/6 Nx + HP diet. A and B: Nx mice, C and D: Nx + HP mice (HE staining, original magnification: × 200). II: Quantification of glomerular cell number, sclerosis index and tubulo-interstitial fibrosis in mice at 12 weeks after Nx or Nx + HP. Values are means ± SEM; *n* = 5 per group. NS = no significant difference vs p53–/– mice.

The morphology of the kidney was normal in groups 1 and 2 eight and 12 weeks after 5/6 Nx (data not shown). The appearance of abnormalities, including glomerular hypertrophy, cellular proliferation, glomerular sclerosis, intratubular casts, tubular dilation and atrophy, and intertubular cell infiltration and matrix accumulation occurred in groups 3 and 4, as well as in groups 5 and 6 at eight weeks, and became progressively worse by week 12. No obvious difference was found when the HP diet was combined with 5/6 Nx compared with 5/6 Nx only (group 5 vs 3 and 6 vs 4, *p* > 0.05), and no significant difference was observed between groups 3 and 4, and groups 5 and 6 at the same time points (Fig. 1).

Compared with sham surgery, 5/6 Nx in both p53+/+ and p53–/– mice caused more than twofold increases in BUN concentrations at eight weeks following 5/6 Nx, which remained elevated throughout the study. Similarly, 5/6 Nx also had significant effects on haemoglobin, plasma Ca, Pi and PTH levels and body weight, but not on mean systolic arterial blood pressure (Table 1). Plasma BUN, haemoglobin, plasma Ca, Pi and PTH levels, body weight and BP were similar in p53+/+ and p53–/– mice after 5/6 Nx (group 3 vs 4), as well as in p53+/+ and p53–/– mice after 5/6 Nx + HP (group 5 vs 6). However plasma Ca, Pi and PTH levels were significantly higher in mice after 5/6 Nx + HP compared with those in mice after 5/6 Nx only (group 5 vs 3 and 6 vs 4, *p* < 0.001 at each time) (Table 1).

**Table 1.** Effects Of 5/6 Nx And Hp Treatment On Body Weight, Blood Pressure And Serum Chemistry In P53+/+ And P53–/– Mice At 12 Weeks

	*p53+/+ mice*			*p53–/– mice*		
	*Sh*	*5/6 Nx*	*5/6 Nx + HP*	*Sh*	*5/6 Nx*	*5/6 Nx + HP*
Body weight (g)	12.1 ± 1.4	17.3 ± 1.7^a^	16.8 ± 2.2^a^	22.6 ± 1.3	18.5 ± 2.1^a^	18.8 ± 2.5^a^
Blood haemoglobin (mmol/l)	10.6 ± 0.6	8.4 ± 0.4^a^	8.2 ± 0.3^a^	10.8 ± 0.5	8.7 ± 0.4^a^	8.6 ± 0.2^a^
Plasma urea (mmol/l)	12.5 ± 1.3	27.7 ± 4.5^b^	29.1 ± 3.8^b^	13.8 ± 2.1	30.2 ± 5.7^b^	32.8 ± 4.2^b^
Plasma calcium (mmol/l)	2.1 ± 0.2	2.8 ± 0.1^a^	3.7 ± 0.2^cd^	2.3 ± 0.2	3.1 ± 0.3^a^	4.1 ± 0.3^cd^
Plasma phosphate (mmol/l)	2.7 ± 0.3	3.6 ± 0.2^c^	4.8 ± 0.4^cd^	3.0 ± 0.4	3.8 ± 0.4^c^	5.0 ± 0.3^cd^
PTH (ng/ml)	67 ± 22	685 ± 221^b^	843 ± 301^be^	72 ± 27	702 ± 237^b^	862 ± 325^be^

Results are expressed as group mean ± SEM at 12 weeks after 5/6 nephrectomy (5/6 Nx) or sham-operation (Sh), *n* = 5 per group. ^a^*p* < 0.05, ^b^*p* < 0.001, ^c^*p* < 0.01 vs untreated Sh. ^d^*p* < 0.01, ^e^*p* < 0.001 vs 5/6 Nx mice. PTH = parathyroid hormone.

## Histopathological analysis of vascular calcification in the aorta

**Fig. 2.**
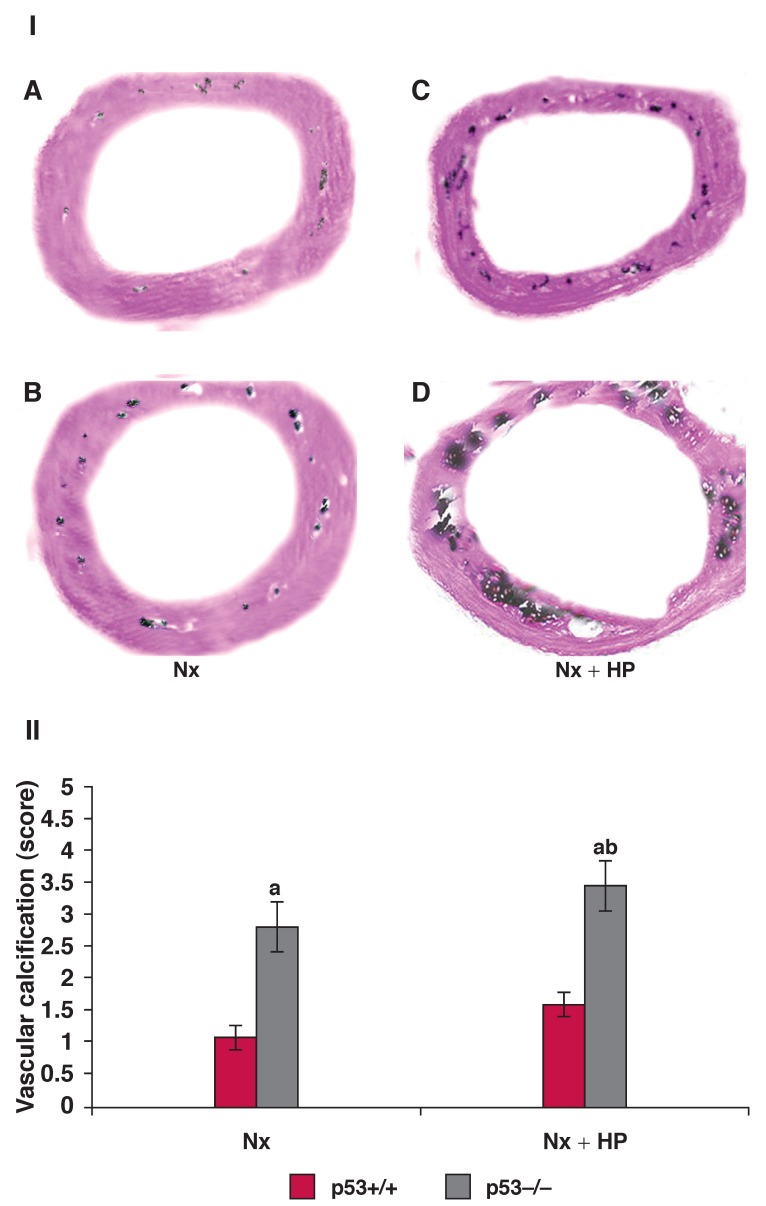
Ⅰ: Histopathological analysis of vascular calcification in the aorta at 12 weeks after Nx or Nx + HP (original magnification, × 200) with von Kossa staining. A and C: p53+/+ mice, and B and D: p53–/– mice. Ⅱ: Evaluation of the vascular calcification score in p53+/+ and p53–/– mice at 12 weeks after 5/6 Nx or 5/6 Nx + HP. Values are means ± SE; *n* = 5 per group. ^a^*p* < 0.001 vs p53+/+ mice, ^b^*p* < 0.01 vs Nx mice.

In groups 1 and 2, no mineral deposition was noted in the 5/6 Nx mice but scattered spots of black-stained material were found in the arterial media layer (Fig. 2A, B) at 12 weeks. In the 5/6 Nx + HP mice, diffuse or massive mineral deposition was detected in the media layer (Fig. 2C, D) at 12 weeks, and the calcification in p53–/– mice was much more severe than in the p53+/+ mice. The aorta in the 5/6 Nx mice had mild/moderate calcification, whereas the aorta in the 5/6 Nx + HP mice, particularly the p53–/– mice, had severe calcification. The score showed that vascular calcification in the p53–/– mice had increased significantly compared with that in the p53+/+ mice. Vascular calcification in the 5/6 Nx + HP mice was also increased compared with that in the 5/6 Nx only mice (Fig. 2).

**Fig. 3.**
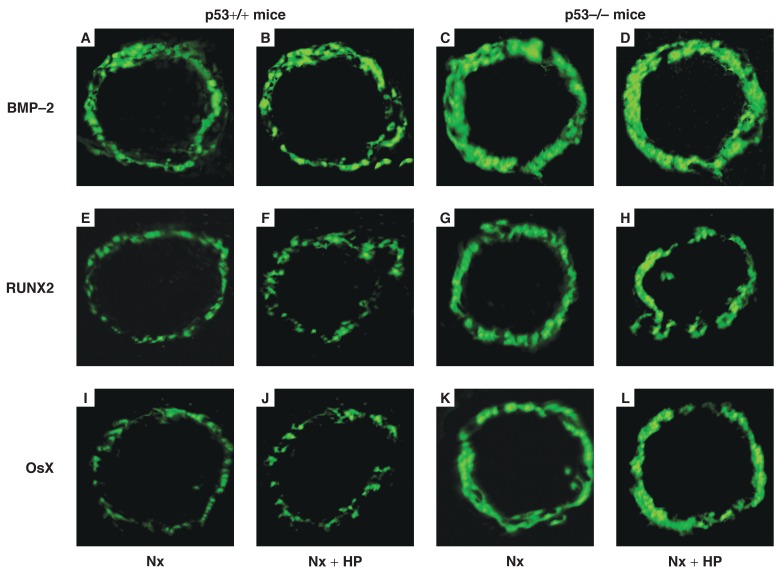
Indirect immunofluorescence staining analysis for BMP-2, RUNX2 and Osx in the aorta at 12 weeks after 5/6 Nx or 5/6 Nx + HP (original magnification, × 200). Positive staining of BMP-2, RUNX2 and Osx protein was detected in VSMCs, clumping or a green line demonstrated the expression of protein, but in p53+/+ or 5/6 Nx mice, a weaker signal was detected compared with those in p53–/– or 5/6 Nx + HP mice.

**Fig. 4.**
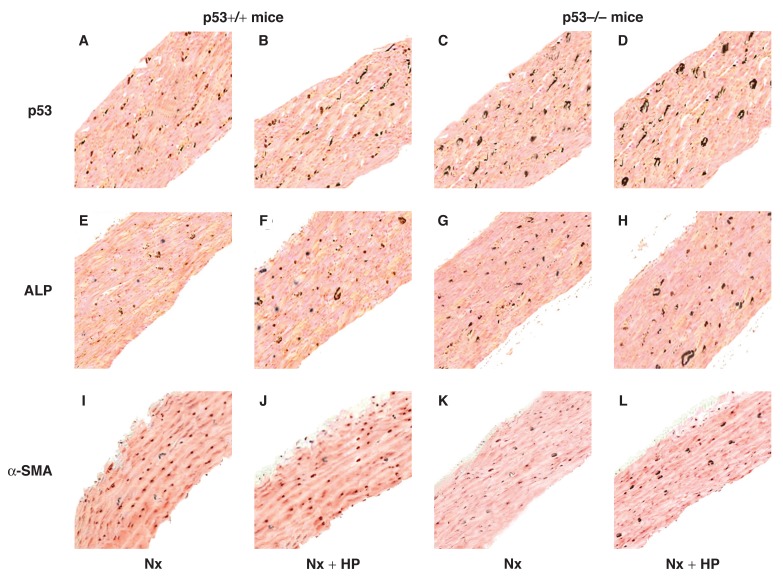
Immunohistochemistry analysis for p53, ALP and α-SMA in the aorta at 12 weeks after 5/6 Nx or 5/6 Nx + HP (original magnification, × 200). In p53–/– mice with 5/6 Nx + HP, ALP expression was high, but α-SMA was low, and no p53 was detected, while related heavy mineral deposition was also detected. On the other hand, a weak positive staining of ALP, and enhanced positive staining of p53 and α-SMA was detected in p53+/+ mice, while moderate mineral deposition could be detected. A brown colour indicated the presence of proteins and a black colour indicated mineral deposition.

**Fig. 5.**
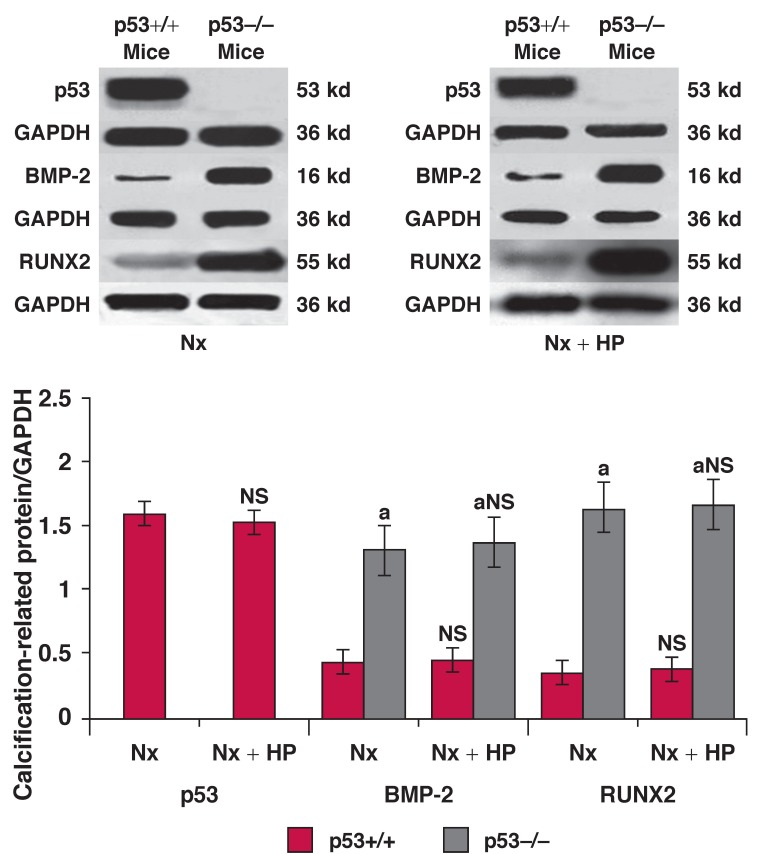
Western blot analysis for p53, BMP-2 and RUNX2 in the aorta at 12 weeks after 5/6 Nx or 5/6 Nx + HP. A densitometry graph shows the expression levels of each calcification-related protein identified by Western blot analysis in the aorta. The graph indicates the relative band density when levels of GAPDH protein expression in each sample were calculated as 100%. Values are means ± SE; *n* = 5 per group. ^a^*p* < 0.001 vs p53–/– mice, NS = no significant difference vs 5/6 Nx mice with the same genetype.

**Fig. 6.**
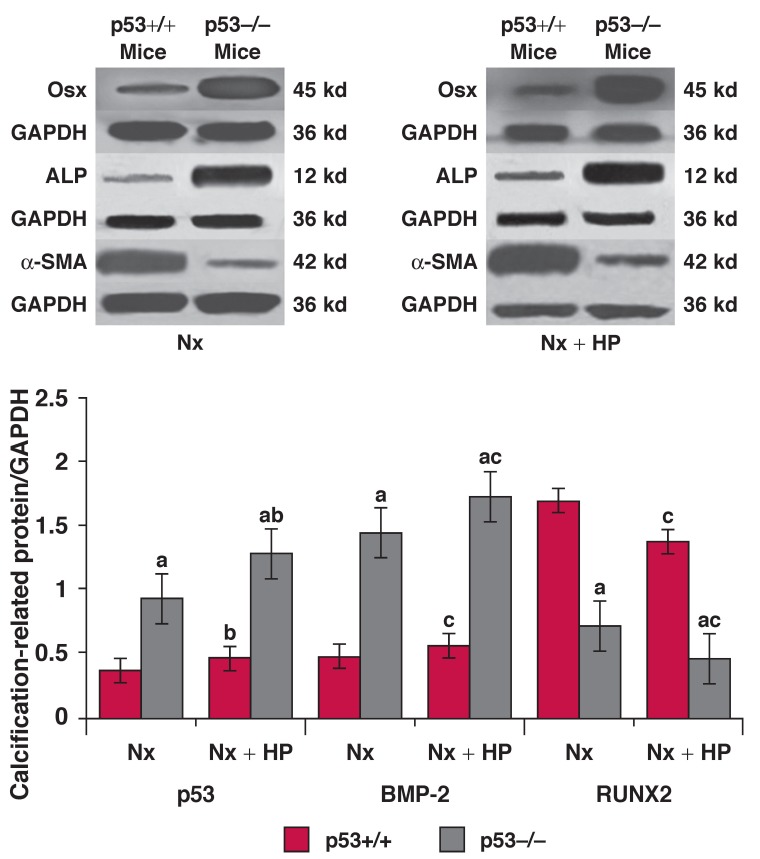
Western blot analysis for Osx, ALP and α-SMA in the aorta at 12 weeks after 5/6 Nx or 5/6 Nx + HP. A densitometry graph shows the expression levels of each calcification-related protein identified by Western blot analysis in the aorta. The graph indicates the relative band density when levels of GAPDH protein expression in each sample were calculated as 100%. Values are means ± SE; *n* = 5 per group. ^a^*p* < 0.001 vs p53–/– mice, ^b^*p* < 0.05, ^c^*p* < 0.01 vs 5/6 Nx mice with the same genetype.

In groups 1 and 2, almost no positive staining of p53, BMP-2, RUNX2, Osx and ALP protein was found in the VSMCs of the aorta, but expression of α-SMA was detected. In group 4 and particularly group 6, positive staining of BMP-2 (Fig. 3C, D), RUNX2 (Fig. 3G, H), Osx (Fig. 3K, L) and ALP protein (Fig. 4G, H) was always detected in the VSMCs. However, no p53 (Fig. 4C, D) and only weak staining of α-SMA (Fig. 4K, L) was detected in the same tissues. This pattern presented at eight weeks after 5/6 Nx, and became significantly increased 12 weeks after 5/6 Nx. On the other hand, weak positive staining of BMP-2 (Fig. 3B, A), RUNX2 (Fig. 3F, E), Osx (Fig. 3J, I) and ALP proteins (Fig. 4F, E), and enhanced positive staining of p53 (Fig. 4B, A) and α-SMA (Fig. 4J, I) was detected in group 5, and particularly in group 3. Quantitative analysis of these proteins was performed using the Western blot analysis, and this showed changes similar to those found by the immunohistochemistry and indirect immunofluorescence analyses (Figs 5, 6).

Statistical analysis showed that changes in vascular calcification were positively correlated with the levels of expression of BMP-2, RUNX2, Osx and ALP, but negatively correlated with the levels of expression of p53 and α-SMA. Correlation coefficients (*R*) and *p*-values are shown in Table 2. The levels of expression of BMP-2, RUNX2, Osx and ALP were significantly negatively correlated with those of p53 and α-SMA. Correlation coefficients and *p*-values are shown in Tables 3 and 4.

**Table 2.** Linear Correlation Between Vascular Calcification And Calcification-Related Proteins In VSMCS After 5/6 Nephrectomy

	*P53(+/+) mice*		*p53(–/–) mice*	
*Protein*	R	p-*value*	R	p-*value*
p53	–0.725	< 0.001		
BMP-2	0.569	< 0.001	0.677	< 0.001
RUNX2	0.457	< 0.001	0.594	< 0.001
Osx	0.510	< 0.001	0.618	< 0.001
ALP	0.670	< 0.001	0.773	< 0.001
α-SMA	–0.582	< 0.001	–0.755	< 0.001

**Table 3.** Linear Correlation Between P53 Protein And Other Calcification-Related Proteins In VSMCS From P53+/+ Mice After 5/6 Nephrectomy

*Protein*	R	p-*value*
BMP-2	–0.671	< 0.001
RUNX2	–0.586	< 0.001
Osx	–0.622	< 0.001
ALP	–0.703	< 0.001

**Table 4.** Linear Correlation Between a-SMA And Other Calcification-Related Proteins In VSMCS After 5/6 Nephrectomy

	*P53(+/+) mice*		*p53(–/–) mice*	
*Protein*	R	p-*value*	R	p-*value*
p53	0.664	< 0.001		
BMP-2	–0.585	< 0.001	–0.629	< 0.001
RUNX2	–0.502	< 0.001	–0.610	< 0.001
Osx	–0.569	< 0.001	–0.716	< 0.001
ALP	–0.742	< 0.001	–0.758	< 0.001

## Discussion

Although circulating calcium and phosphate concentrations have been correlated with the progression of vascular calcification in longitudinal studies of dialysis patients,^22^,^23^ they only partly account for the calcification. It is clear then that vascular calcification is controlled by factors other than circulating calcium and phosphate levels.

In a previous study, we found that the expression of p53 was inhibited in VSMCs from patients on maintenance haemodialysis, while vascular calcification was widely present.^13^ In line with these findings, Ohmi *et al*.^24^ found that VSMCs from p53 knock-out mice aortas revealed an extended bipolar shape and expressed h-caldesmon and calponin, as well as a smooth muscle actin as protein markers of differentiated smooth muscle. This implies that p53–/– VSMCs have the potential to transform to osteoblast-like cells, and that p53 may inhibit the transformation of VSMCs to osteoblast-like cells. We therefore propose that the inhibition of expression of p53 in VSMCs may be involved in the pathogenesis of the osteogenic differentiation of VSMCs in CKD.

To test this hypothesis, we used the experimental model of CKD-associated vascular calcification in p53+/+ and p53–/– mice with 5/6 Nx and HP diet. At eight and 12 weeks after 5/6 Nx, aortic calcification, and markers of osteogenic differentiation, smooth muscle-specific protein and p53 proteins in the aortic tissue were studied, and the effects of p53 on osteogenic differentiation of VSMCs were assessed.

We found that changes in kidney histopathology and plasma BUN levels showed no significant difference between the p53+/+ and p53–/– mice at eight and 12 weeks following 5/6 Nx, which indicates that both p53+/+ and p53–/– mice developed to the same stage of CKD at the same time following 5/6 Nx. There were no obvious differences in deposition of serum calcium phosphate between groups 1 and 2, groups 3 and 4, and groups 5 and 6, which indicated that the disruption by p53 had no effect on the calcium–phosphate homeostasis.

In sham-operated mice, mineral deposition was not assessed. In p53+/+ mice with 5/6 Nx, and particularly with 5/6 Nx + HP, scattered mineral deposition was found in the arterial media layer. In p53–/– mice with 5/6 Nx, and particularly with 5/6 Nx + HP, diffuse or large-scale mineral deposition was detected in the media layer. Therefore the aortas of p53+/+ and p53–/– mice with 5/6 Nx had mild/moderate calcification, and the aortas of p53–/– mice with 5/6 Nx + HP had severe calcification.

The vascular calcification score showed that in p53–/– mice and in mice with 5/6 Nx + HP, vascular calcification was significantly increased compared with that in p53+/+ mice and in mice with 5/6 Nx only. This result suggests that (1) the vascular calcification may be inhibited by p53 in CKD mice, and (2) the 5/6 Nx plays a vital role in vascular calcification, and the HP diet aggravates vascular calcification.

Our further investigation showed that almost no positive staining of p53, RUNX2, Osx and ALP proteins was found in the VSMCs from sham-operated mice, but expression of α-SMA was detected. In p53–/– mice, positive-staining of RUNX2, Osx and ALP proteins was detected in VSMCs, but no p53 and only weak staining of α-SMA was detected in the same tissue. This pattern presented at eight weeks after 5/6 Nx, and became more apparent at 12 weeks after 5/6 Nx. On the other hand, weak positive staining of RUNX2, Osx and ALP proteins, and enhanced positive staining of p53 and α-SMA was detected in p53+/+ mice.

Statistical analysis indicated that changes in vascular calcification were positively correlated with levels of expression of RUNX2, Osx and ALP, but negatively correlated with levels of expression of p53 and α-SMA. Levels of expression of RUNX2, Osx and ALP were significantly negatively correlated with levels of expression of p53 and α-SMA. These results indicate that p53 has an inhibitory effect on osteogenic differentiation of VSMCs in CKD mice, as evidenced by an increase in markers of osteogenic differentiation and a decrease in expression of the smooth muscle-specific marker. However, the mechanisms by which p53 inhibited osteogenic differentiation of VSMCs were not clear.

We then investigated the possible mechanisms whereby p53 inhibited osteogenic differentiation of VSMCs in CKD mice. Previous studies suggest that vascular calcification and bone formation may share common regulatory mechanisms.^25^-^28^ In bone, BMP-2 promotes osteoblast differentiation and mineralisation. BMP-2 is a potent osteogenic protein required for osteoblast differentiation and bone formation, which has been implicated in vascular calcification.^25^ BMP-2 promoted transition of the osteochondrogenic phenotype of VSMCs, also evidenced by an increase in marker expression of RUNX2 and a decrease in marker expression of VSMC.^25^

Runx2 is a transcription factor critical for osteogenesis and bone formation and is expressed during ectopic vascular calcification. A study by Tanaka *et al*.^29^ demonstrated that RUNX2 could repress myocardin-induced differentiation and concomitantly promote the osteogenic conversion of VSMCs. The expression level or activity of the RUNX2 protein is critical for the osteoblastic differentiation of VSMCs.

Osx is genetically downstream of RUNX2.^30^ The studies by Wang *et al*.^31^ and Lengner *et al*.^12^ provide compelling evidence that p53 suppresses osteoblast differentiation by repressing the expression of either RUNX2 or Osx. The subtle discrepancy that exists between the two studies (whether RUNX2 or Osx is the target of p53 action) may be related to how p53 activity is targeted and whether this mechanism alters the stage of cell differentiation. In either case, the concept is that the absence of a tumour-suppressor gene can enhance cell proliferation while favouring differentiation.^30^

Fujita *et al*.^32^ found that BMP-2 could induce new bone formation *in vivo* by the BMP–p53–Cbfa1–Osterix axis in the osteoblast lineage. p53 is a negative regulator of Osx, and osteoblasts deficient in p53 exhibited an enhanced ability to promote osteoblast-dependent osteoclastogenesis.^32^

In our study, no expression of BMP-2 was found in the VSMCs from sham-operated mice, but in the VSMCs from p53–/– mice, positive staining of BMP-2 proteins was increased along with the increase in expression of RUNX2, Osx and ALP. Conversely, positive staining of BMP-2 proteins was decreased along with the decrease in expression of RUNX2, Osx and ALP in p53+/+ mice. The level of expression of BMP-2 correlated negatively with that of p53, while α-SMA correlated negatively with the level of expression of calcification-related proteins and positively with that of p53 (Tables 2, 3, 4).

From the data above, our results imply that p53 may repress osteogenic differentiation of VSMCs in CKD by inhibiting the expression of BMP-2 and/or RUNX2 and Osx protein directly. This indicates that the BMP–p53–Cbfa1(RUNX2)–Osterix axis in osteoblast-dependent osteoclastogenesis is involved in the mechanism of osteogenic differentiation of VSMCs in CKD mice.

Clinically, a percentage of CKD patients in dialysis do not have vascular calcification and also do not show development or progression of calcification, implying that there are most likely genetic factors that predispose to protection from vascular calcification. p53 may play a role in these patients. The precise molecular targets of p53 in these mechanism remains to be elucidated in further *in vitro* study.

## Conclusion

We found that a p53 deficiency resulted in phenotype changes and elevated phosphate-induced mineralisation in VSMCs from 5/6 Nx mice. The effect of p53 repressing osteogenic differentiation of VSMCs is most likely to be mediated in part by down-regulation of BMP-2 expression and/or expression of RUNX2 and Osx directly. This study has provided a possible novel mechanism to show that p53 could be a negative regulator of osteogenic differentiation of VSMCs in CKD mice. It raises an interesting question regarding future therapeutic strategies for vascular calcification in CKD patients by up-regulating the expressson of p53.
